# Hepatic Tumoral Calcinosis in a Patient With Obesity and Metabolic Syndrome: A Case Report

**DOI:** 10.1155/cris/9210483

**Published:** 2026-11-01

**Authors:** Tianshi Xiang, Fei Xie, Mengmeng Ji, Zheng Liu, Feng Jiang, Xiaoping Liu

**Affiliations:** ^1^ The First Clinical Medical College of Gannan Medical University Ganzhou China; ^2^ Ganzhou City Key Laboratory of Colorectal and Anal Diseases Research Ganzhou China; ^3^ Department of General Surgery The First Affiliated Hospital of Gannan Medical University Ganzhou China gmu.cn

**Keywords:** calcium hydroxyapatite, liver, liver tumour, robotic surgery, tumoral calcinosis

## Abstract

Tumoral calcinosis is a kind of non‐tumour calcium salt deposition, is an extremely rare disease, is also a soft tissue rare non‐tumour lesion, belongs to the autosomal dominant genetic disease, mostly occurring in adolescents, seldom exceeding the age of 50 years, with a similar proportion of men and women, and its tumoral calcinosis occurs in the bone and joint parts, especially located in the femur rotator cuff, the elbow, the lateral part of the shoulder and the hip parts, which is manifested as it is characterised by painless subcutaneous calcified masses around the joints, which slowly increase in size, with a history of up to several years, and generally without obvious clinical symptoms. Therefore, this disease usually occurs in the bone and joints, and less frequently in the parenchymal organs. In this paper, we present a case of tumoral calcinosis in the liver, in which the patient was found to have a 24‐year‐old male presented with a 9‐day history of a space‐occupying lesion in the left lateral segment of the liver adjacent to the gastric curvature, accompanied by vague abdominal pain on satiety, without nausea, vomiting, chills, fever, or jaundice. Imaging features, laboratory tests and pathological findings confirmed our diagnosis, and a good outcome was achieved by surgical resection of tumoral calcinosis in the liver.

## 1. Introduction

Tumoral calcinosis is non‐neoplastic lesions that appear as tumour‐like hyperdense shadows on imaging CT, most commonly at the osteoarticular sites. Tumoral calcinosis is a very rare disease; pathologically, calcium hydroxyapatite is the main component of the calcium salt deposits, which are mixed with histiocytes, macrophages, multinucleated giant cells and lymphocytes, and may be surrounded by fibrotic tissue encapsulation or inflammatory reaction [1–4]. Tumoral calcinosis occurring in the liver is much rarer. Therefore, hepatic tumoral calcinosis is a rare pathology characterised by the presence of tumour‐like foci of calcium salt deposition in the liver, which mainly manifests as focal or diffuse tumoral calcinosis in the liver and may be misdiagnosed as a hepatic tumour by imaging [5]. Tumoral calcinosis in the liver is usually asymptomatic and is usually detected by unintentional physical examination. If the size is too large, it may cause the same nonspecific symptoms as hepatocellular carcinoma: Mild abdominal pain and abdominal distension in the right upper abdomen, and if the tumour is too large, it may compress the bile ducts or blood vessels, causing jaundice or portal hypertension [6]. Here, we report a case of tumoral calcinosis in the liver.

## 2. Case Presentation

A 24‐year‐old male presented with a 9‐day history of a space‐occupying lesion in the left lateral segment of the liver adjacent to the gastric curvature, accompanied by vague abdominal pain on satiety, without nausea, vomiting, chills, fever, or jaundice. The rest of the patient’s condition was unremarkable, and there was no family history of thyroid carcinoma or other malignancies.

On physical examination, the abdomen was distended and slightly hard, with no tenderness, rebound tenderness, or palpable abdominal mass. No jaundice was observed on the skin or sclera, no palmar erythema, no spider angiomas, and no ascites were detected. The liver and spleen were not palpable below the costal margin; shifting dullness was negative, bowel sounds were normal, and no percussion pain was elicited in either renal region. The patient’s admission weight was 83 kg with a height of 165 cm, resulting in a BMI of 30.49 kg/m^2^.

Preoperative laboratory tests revealed: Alanine aminotransferase (ALT) 166 U/L, aspartate aminotransferase (AST) 62 U/L, albumin (ALB) 43.8 g/L, and glutathione reductase (GR) 102 U/L. The lipid profile indicated mixed hyperlipidaemia: Total cholesterol (CHO) 5.8 mmol/L, high‐density lipoprotein (HDL) CHO 0.86 mmol/L, low‐density lipoprotein (LDL) CHO 3.68 mmol/L, and non‐HDL CHO 4.94 mmol/L. The patient was placed on a low‐fat diet and received lipid‐lowering medication prior to surgery. Serum phosphorus level was 0.96 mmol/L (normal range: 0.81–1.45 mmol/L), which was within the normal range, and no specific phosphate‐lowering therapy was required. The elevated liver enzymes (ALT and AST) were consistent with fatty liver‐related hepatic injury.

Contrast‐enhanced CT revealed a mass‐shaped high‐density focus in the left lateral segment of the liver, measuring ~62 × 33 mm, with a marginal CT value of about 1500 HU and no obvious enhancement on the contrast scan, suggestive of a benign lesion such as calcinosis (Figure 1). Coronal contrast‐enhanced CT images further demonstrated that the lesion was contiguous with the liver parenchyma and adjacent to the stomach without direct invasion (Figure 2). Conclusion: 1. Para‐occupation of the left lateral segment of the liver, benign possibility; 2. Fatty liver with parasplenic nodule (consistent with accessory spleen); 3. Bilateral pulmonary infection: Recommend follow‐up.

**Figure 1:**
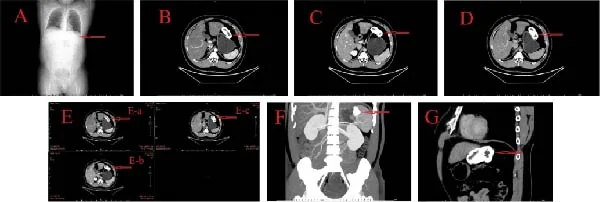
(A) Abdominal plain film, the arrow shows a mass‐shaped hyperdense focus next to the left lateral segment of the liver. (B) Arterial phase of whole‐abdomen enhancement scan, the arrow shows a mass‐shaped hyperdense focus next to the left lateral segment of the liver, with clear borders and low‐density areas, and no significant enhancement was seen in the enhancement scan. (C) Portal phase of whole‐abdomen enhancement scan, the arrow shows a mass‐shaped hyperdense focus next to the left lateral segment of the liver, with clear borders and low‐density areas. (D) Delayed phase of whole‐abdomen enhancement scan, the arrow shows a mass‐shaped high‐density focus next to the left lateral segment of the liver, with clear borders, low‐density areas and no obvious attenuation of the high‐density areas. (E) CT image of the arterial phase, portal vein phase and delayed phase in the same plane. E‐a corresponding to the arterial phase in (B); E‐b corresponding to the portal venous phase in (C); and E‐c corresponding to the delayed phase in (D). (F) The arrow shows a mass‐shaped hyperdense focus next to the left lateral segment of the liver. (G) The arrow shows a mass‐shaped hyperdense focus next to the left lateral segment of the liver.

**Figure 2:**
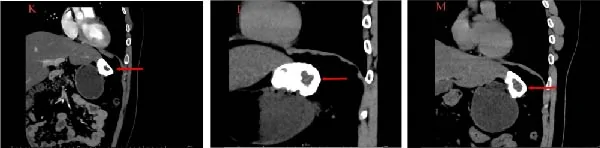
(K, L, M): Coronal contrast‐enhanced CT images demonstrating that the hepatic calcinosis lesion is contiguous with the liver parenchyma and adjacent to the stomach without direct invasion.

Problem List: 1. Space‐occupying lesion in the left outer quadrant of the liver; 2. Obesity; 3. Mixed hyperlipidaemia; 4. Pulmonary infection; 5. Fatty liver.

Given the patient’s comorbidities of obesity, fatty liver and elevated liver enzymes, we could not completely rule out the possibility of malignancy despite the benign imaging features. Preoperatively, we considered performing ultrasound‐guided percutaneous biopsy; however, we were concerned about the potential risk of needle tract seeding and tumour dissemination if the lesion proved to be malignant. After thorough discussion with the patient regarding the risks and benefits, the patient expressed a clear preference for direct surgical resection without prior biopsy. Therefore, surgical resection was indicated for both definitive pathological diagnosis and therapeutic purposes. In addition, the patient’s BMI of 30.49 kg/m^2^ with obesity, metabolic syndrome, hyperlipidaemia and fatty liver met the indications for bariatric surgery. To avoid a second anaesthesia and second hospitalisation, we decided to perform concurrent robotic‐assisted resection of the hepatic lesion and robotic‐assisted sleeve gastrectomy.

Under general anaesthesia, the patient was placed in the supine position, and the surgical field was routinely prepared and draped. Exploring the abdominal cavity, a hard, greyish‐white mass (~7 × 2 cm in size) was identified adhering to the margin of the left lateral segment of the liver, with partial adhesion to the greater omentum. Intraoperative exploration confirmed that the lesion was directly contiguous with the hepatic parenchyma, with well‐defined borders and no obvious adhesion to the gastric wall (Figure 3). The robotic ultrasonic knife was used to detach the adherent omental tissue and gradually free the tumour along the margin of the left lateral segment, with ligation of the intrahepatic bile ducts, arteries and portal vessels until the tumour was completely resected, and the hepatic section was thoroughly hemostatized. Robot‐assisted sleeve gastrectomy was performed concurrently, following standard surgical techniques.

**Figure 3:**
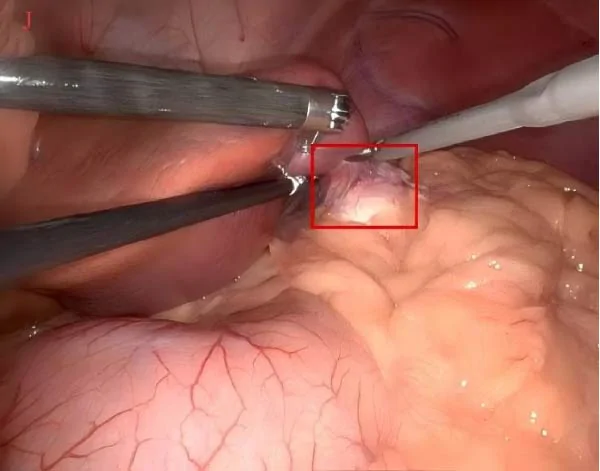
Intraoperative photograph of the hepatic lesion. The greyish tissue within the red square represents the tumoral calcinosis lesion, which can be clearly observed to be directly connected to the liver parenchyma, with no contact with the gastric wall.

Given the benign appearance on both preoperative imaging and intraoperative gross inspection, intraoperative frozen section was not performed. Moreover, the frozen section would not have altered the surgical plan as the lesion required complete resection for definitive diagnosis. The specimen was removed, and the operation was completed without complications.

Postoperatively, the patient received anti‐infection and acid‐suppression therapy, was encouraged to ambulate early, and received enteral nutritional support. Gross examination of the resected hepatic specimen revealed a grey‐yellow and grey‐red tissue measuring 8.5 × 5 × 3.5 cm, with a grey‐yellow, hard‐cut surface and a central cavity containing grey‐yellow fluid. Microscopic examination revealed extensive calcium salt deposits surrounded by fibrous collagen, with vasodilatation and congestion. At the interface between the lesion and the adjacent hepatic parenchyma, a clear transition zone was observed, where calcified deposits gradually merged with the surrounding liver tissue. The residual liver tissue at the margins showed hepatocyte oedema with steatosis and focal lymphocytic infiltration. Examination of the gastric specimen revealed focal lymphocytic infiltration and lymphoid follicle formation in the proximal gastric tissue (Figures 4 and 5). Based on biochemical examination, imaging findings and pathological examination, without evidence of ectopic calcification of other secondary diseases, the final diagnosis was primary normophosphatemic tumoral calcinosis.

**Figure 4:**
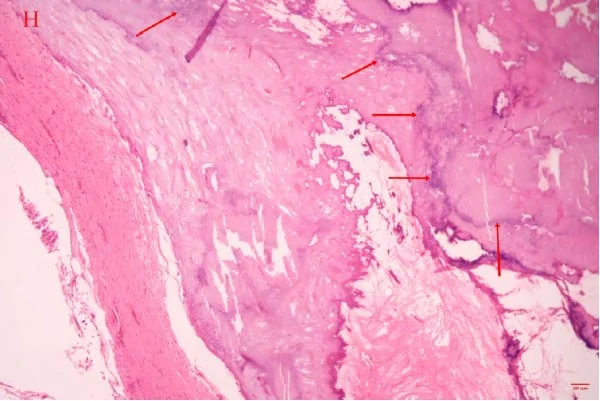
Histopathological examination of the hepatic lesion. The image (H) shows extensive calcium salt deposits surrounded by fibrous collagen with vasodilatation and congestion, with residual liver tissue at the margins. The red arrows indicate the large areas of dark purple amorphous basophilic regions, representing the core pathological feature of calcium deposition within the hepatic parenchyma (scale bar: 200 µm).

**Figure 5:**
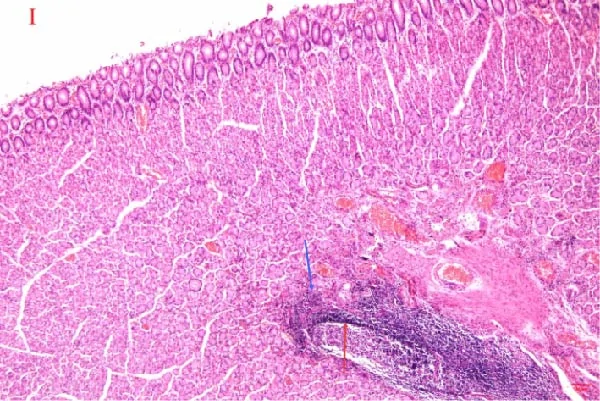
Histopathological examination of the gastric resection specimen (I). The blue arrows indicate focal lymphocytic aggregates within the lamina propria of the gastric mucosa, and the red arrows point to the densely cellular lymphoid follicles (dark purple areas) (scale bar: 200 µm).

Postoperative follow‐up showed no significant abnormality, and a repeat abdominal CT scan confirmed that the hepatic tumoral calcinosis had resolved without recurrence.

## 3. Discussion

Tumoral calcinosis is an idiopathic soft tissue calcium salt deposition disorder that occurs in the vicinity of large joints but does not involve the synovial membrane; tumoral calcinosis is a rare disease also known as tumour‐like calciphylaxis, calcified collagenolysis and calcified pseudotumour [7–9]. In contrast, hepatic tumour‐like calciphylaxis is an idiopathic soft‐tissue calcium salt deposition disease occurring in the liver, in which calcium hydroxyapatite is the main component of the calcium salt deposits, and it appears as a hyperdense shadow on CT imaging. Tumoral calcinosis can occur in healthy children and young adults, and it is characterised by asymptomatic, calcified and massive masses [10].

In adolescence, tumoral calcinosis occurs in males, while in adulthood, it occur in females [3]. The first cases of tumoral calcinosis were reported by two French authors, Giard and Duret in 1898 and 1899 [1, 11, 12], and Inclan et al. reported this case in 1943 and used the term ’tumoral calcinosis’ for the first time, which provided the basis for subsequent reports on the subject [13]. In 1969, McClatchie reported 26 cases of tumoral calcinosis, which were often found around the hip and buttocks, in muscles and subcutaneous tissues and rarely in viscera, and in this paper, we report a case of tumoral calcinosis in the liver, which is even more extremely rare [14].

Foreign scholars Smack et al., through a review of 121 cases of tumour calcification, systematically derived three subtypes of tumoral calcinosis: (1) primary normophosphatemia tumoral calcinosis: The patient has no evidence of previous disease associated with soft tissue calcification, the patient’s serum phosphate and serum calcium are normal, and the patient’s body appeared in tumoral calcinosis: (2) primary hyperphosphatemia tumoral calcinosis: The patient’s body appeared in tumoral calcinosis, and the patient’s body was very rare. Calcitoninosis: The patient has no evidence of prior disease associated with soft tissue calcification, the patient has elevated serum phosphorus levels but normal serum calcium, and the patient develops tumoral calcinosis: (3) secondary tumour calciphylaxis: The patient has a disease that can lead to soft tissue calcification, and subsequently, the patient develops tumoral calcinosis [15].

Slavin et al. [4] systematically discussed the risk of tumoral calcinosis in patients who present with the cause of tumoral calcinosis, due to elevated serum phosphorus levels, which promote the nucleation of hydroxyapatite, collagen, osteoclast cytoplasm, matrix lipid fragments in lysosomes, tubular contents and other extracellular matrix materials deposited in mitochondria; the inability of tumoral calcinosis to pass through the mesangial cells and osteoclasts, which further promotes calcium salt deposition, and the inability of hydroxyapatite to be dissolved by these cells, resulting in tumoral calcinosis cells, resulting in the formation of tumoral calcinosis.

In another report of tumoral calcinosis in dermatomyositis, it was mentioned that the patient’s blood calcium, alkaline phosphate, 25‐hydroxyvitamin D, parathyroid hormone and renal function tests were normal, and the deposit was shown by laboratory tests to be calcium phosphate, i.e., carbonate apatite: [Ca_10_(PO_4_CO_3_OH)_6_(OH)_2_] [16]. In reports of Auraemic tumoral calcinosis and chronic lupus nephritis tumoral calcinosis, the similarities were that both required prolonged dialysis, and the histopathology of the deposits was suggestive of basophilic calcium phosphate deposits and amorphous calcium deposits, but the patients both suggested the presence of hyperparathyroidism [17, 18]. In contrast, in a Japanese case of uraemic tumoral calcinosis, the patient was shown to have increased blood urea nitrogen, creatinine, parathyroid hormone, calcium and phosphorus [19].

A foreign review detailed that hyperphosphatemic familial tumoral calcinosis deposition (HFTC) is caused by a disorder of fibroblast growth factor 23 (FGF23)‐mediated regulation of phosphate and that the disease manifests itself as hyperphosphatemia, ectopic calcification and abnormally increased tubular reabsorption of phosphate and 1,25‐dihydroxyvitamin D [20]. Hyperphosphatemic familial tumoral calcinosis is caused by mutations in three bi‐allelic genes: Type 1 for GALNT3, type 2 for FGF23 and type 3 for α‐Klotho, with reduced FGF23 secretion arising from mutations in the FGF23 or GALNT3 genes, which are involved in phosphate metabolism [21]. This suggests that risk factors predisposing to hepatic tumoral calcinosis may include female gender, hyperphosphatemia, hypercalcaemia, elevated calcium‐phosphorus product, increased calcium phosphate products, hyperparathyroidism and increased intake of active vitamin D [18, 22]. Tumoral calcinosis occurs at the osteoarticular sites, spine, etc. [1–3, 11, 12, 14, 23–27], and tumoral calcinosis has also been documented in dermatomyositis [16], but tumoral calcinosis occurring in the liver is rare. Tumoral calcinosis in the liver appear on CT as a mass of dense foci with clear borders and no significant enhancement on enhancement scans, similar to hepatic calcification [28].

Preoperative hepatic tumoral calcinosis is mainly diagnosed by imaging CT, but they still need to be compared with calcified foci of hepatic sarcoidosis, calcium deposits of hepatic encapsulation, calcified foci of hepatic haemangiomas, portal vein thrombosis, hepatic schistosomiasis, hepatocellular adenomas, focal nodular hyperplasia of the liver, epithelioid haemangioendotheliomas of the liver, calcified foci of hepatocellular carcinoma, calcified foci of intrahepatic cholangiocarcinoma, hepatic metastasis tumours, intrahepatic cholangiocarcinoma with mucinous tumours, focal lesions and pseudo lesions in hepatic steatosis, gallstones and hepatic haemorrhage, among other related conditions [29, 30]. Tumoral calcinosis in the liver does not have specific clinical symptoms, and is usually detected by physical examination; if they are too large, they may cause the same nonspecific symptoms as hepatocellular carcinoma: Mild abdominal pain and distension in the right upper abdomen, jaundice, portal hypertension and ascites, but there are generally no risk factors for hepatocellular carcinoma [31]. Tumoral calcinosis is divided into primary normophosphatemic tumoral calcinosis, primary hyperphosphatemic tumoral calcinosis and secondary tumour calcinosis [15].

Treatment of primary normophosphatemic tumoral calcinosis may be better by surgical resection of the tumour because postoperative tumour recurrence is relatively rare; for the treatment of primary hyperphosphatemic tumoral calcinosis, the first step is to have a low‐phosphorus diet, which can be used with a phosphate‐binding agent, which can reduce serum phosphorus to prevent postoperative tumoral calcinosis recurrence, and also with acetazolamide, which is a carbonic anhydrase inhibitor, which acidifies the renal tubules, thus increasing the urinary excretion of phosphate and therefore may reduce phosphate levels [32].

In this paper, hepatic tumoral calcinosis is rare and treatment modalities need to be explored; if the patient is primary hyperphosphatemia, tumoral calcinosis treatment modalities may consider surgical treatment combined with oral acetazolamide, a carbonic anhydrase inhibitor, which is beneficial in reducing the size of the tumour [33]. This case is unique in that the patient also suffered from hyperlipidaemia and fatty liver with pathology suggestive of concomitant fat necrosis. Hyperlipidaemia may also promote the formation of hepatic tumoral calcinosis, which is primary normophosphatemic tumoral calcinosis, and surgical treatment is the preferred first‐line treatment modality for hepatic tumoral calcinosis in the quiescent stage, with a low probability of recurrence [34]. In contrast, for secondary tumour calciphylaxis, resolution of the cause of metabolic dysfunction is preferred.

## Author Contributions

**Tianshi Xiang:** conceptualisation, investigation, writing – original draft, writing – review and editing. **Fei Xie:** investigation, data curation, writing – review and editing. **Mengmeng Ji:** formal analysis, validation, writing – review and editing. **Zheng Liu:** methodology, resources, supervision, writing – review and editing. **Feng Jiang:** supervision, project administration, writing – review and editing. **Xiaoping Liu:** funding acquisition, supervision, writing – review and editing.

## Funding

This work was supported by Ganzhou City Key Laboratory of Colorectal and Anal Diseases Research (Grant 20220101‐25).

## Disclosure

This article has not been published in any journal. All authors of this article have read and approved the final manuscript.

## Consent

This article involves some recognisable images/information of the patient; therefore, we sought the patient’s own opinion and obtained written consent from the patient, who consented to the publication of this case in Case Reports in Surgery, which was signed by the patient.

## Conflicts of Interest

The authors declare no conflicts of interest.

## Data Availability

No datasets were collected or analysed during the preparation of this case report. All relevant clinical and diagnostic data supporting the findings are included within the manuscript.
